# How to be patient. The ability to wait for a reward depends on menstrual cycle phase and feedback-related activity

**DOI:** 10.3389/fnins.2014.00401

**Published:** 2014-12-09

**Authors:** Luise Reimers, Christian Büchel, Esther K. Diekhof

**Affiliations:** ^1^Neuroendocrinology Unit, Institute for Human Biology, Biocenter Grindel and Zoological Museum, University of HamburgHamburg, Germany; ^2^Department of Systems Neuroscience, University Medical Center Hamburg-EppendorfHamburg, Germany

**Keywords:** menstrual cycle, estradiol, dopamine, time perception, fMRI, RCZ, VMPFC, reinforcement learning

## Abstract

Dopamine (DA) plays a major role in reinforcement learning with increases promoting reward sensitivity (*Go learning*) while decreases facilitate the avoidance of negative outcomes (*NoGo learning*). This is also reflected in adaptations of response time: higher levels of DA enhance speeding up to get a reward, whereas lower levels favor slowing down. The steroid hormones estradiol and progesterone have been shown to modulate dopaminergic tone. Here, we tested 14 women twice during their menstrual cycle, during the follicular (FP) and the luteal phase (LP), applying functional magnetic resonance imaging while they performed a feedback learning task. Subsequent behavioral testing assessed response time preferences with a clock task, in which subjects had to explore the optimal response time (RT) to maximize reward. In the FP subjects displayed a greater learning-related change of their RT than during the LP, when they were required to slow down. Final RTs in the slow condition were also predicted by feedback-related brain activation, but only in the FP. Increased activation of the inferior frontal junction and rostral cingulate zone was thereby predictive of slower and thus better adapted final RTs. Conversely, final RT was faster and less optimal for reward maximization if activation in the ventromedial prefrontal cortex was enhanced. These findings show that hormonal shifts across the menstrual cycle affect adaptation of response speed during reward acquisition with higher RT adjustment in the FP in the condition that requires slowing down. Since high estradiol levels during the FP increase synaptic DA levels, this conforms well to our hypothesis that estradiol supports *Go learning* at the expense of *NoGo learning*. Brain-behavior correlations further indicated that the compensatory capacity to counteract the follicular *Go bias* may be linked to the ability to more effectively monitor action outcomes and suppress bottom-up reward desiring during feedback processing.

## Introduction

Time estimation is an essential perceptual skill involved in a variety of behavioral aspects. It influences our decision making in every-day life such as which line to choose at the supermarket based on previous experiences, but also plays a role in motor control (e.g., speed of speech or movement). As to the mechanism underlying time perception in the seconds-to-minutes range, also known as interval timing, a model of an internal clock has been proposed. According to this model a pacemaker sends pulses representing units of elapsed time to a counter, which thereby obtains an estimate of a time duration (Treisman, 1963). However, the speed of this pacemaker is prone to outer influences and thus only reflects a subjective and possibly inaccurate perception of time. More precisely, a higher frequency of sent pulses by the pacemaker results in an overestimation of passed time. For instance, an emotionally arousing context or stimulating substances such as cocaine or methamphetamine may increase the speed of the inner clock (Maricq and Church, 1983; Matell et al., 2004; Droit-Volet and Gil, 2009). Likewise, evidence from animal studies indicates an important role of the steroid hormone 17ß-estradiol (E2) in the modulation of time perception. Accordingly, ovariectomized rodents displayed enhanced internal clock speed after E2 injection (Sandstrom, 2007; Pleil et al., 2011). However, E2 may not directly modulate internal clock speed, but its effect on time perception may rather be a function of the interaction between E2 and dopamine (DA), a neurotransmitter that has also been implicated in time estimation (Rammsayer, 1993; Meck et al., 1996; Buhusi and Meck, 2005). This idea is also supported by other rodent studies reporting a modulating role of E2 on mesolimbic dopaminergic transmission. E2 thereby enhanced dopaminergic tone, on the one hand by stimulating DA release and DA synthesis rate and on the other hand by increasing the density of DA D1 receptors and downregulating D2 receptors (Lévesque et al., 1989; Becker, 1999). In contrast to that, the neurosteroid progesterone (PROG) opposes these effects. PROG has been shown to diminish E2 receptor density, to stimulate enzymes involved in the degradation of DA, and to act positively on GABA receptors, thereby inhibiting dopaminergic neurons, all of which leading to a reduced dopaminergic tone (Luine and Rhodes, 1983; Dluzen and Ramirez, 1984; Majewska et al., 1986; Mauvais-Jarvis et al., 1986). In that way, it may be hypothesized that these neurohormones should exert contrasting effects on the perception of time by increasing vs. reducing internal clock speed.

DA has been proposed to act on action selection via a direct “Go” or an indirect “NoGo” projection pathway from the basal ganglia to the cortex by either facilitating responses or by inhibiting them respectively (Frank et al., 2004). Both, the D1 and D2 receptor type, are integrated in these pathways and stimulated differently by prevalent DA levels. Frank et al. (2004) found that during a reinforcement learning task Parkinson patients being off medication, who thus suffered from depleted DA levels, showed an impaired ability to learn via the “Go” pathway to choose a rewarded stimulus. At the same time, they displayed increased punishment sensitivity and were more able to avoid stimuli that would have led to a negative feedback (i.e., they exhibited better learning via the “NoGo” pathway). The authors proposed that the DA bursts signaling a reward, facilitate learning via positive reinforcement by acting on D1 receptors in the “Go” pathway. On the other hand dips in DA levels caused by negative feedback stimulate the D2 receptor type, which is implemented in the indirect “NoGo” pathway (Frank et al., 2004). This principle has also been shown to transfer to the response time domain: Parkinson patients on medication (i.e., with normal DA levels) were better in learning to adapt their response time to a high response speed in order to maximize their reward. However, when being off medication (i.e., decreased DA levels) they showed better performance in a condition, in which they had to slow down and wait to get the highest reward (Moustafa et al., 2008). Hence, the adaptation of response speed in the context of maximizing reward value appears to be also regulated by the two different pathways, in that the “Go” pathway favors speeding up and the “NoGo” pathway facilitates slowing down.

In our study, we wanted to investigate (1) whether the preference for “Go” over “NoGo” learning in a response time adjustment paradigm depends on naturally varying levels of E2 and PROG during the menstrual cycle and (2) whether the ability to wait for a reward depends on neural responses during reinforcement learning. For this purpose, we tested healthy female subjects twice during their menstrual cycle: once during the late follicular phase (FP), in which E2 levels are high and PROG levels low, and a second time during the mid luteal phase (LP) that is dominated by increased PROG levels. On both test days, subjects underwent functional magnetic resonance imaging (fMRI) while performing a probabilistic feedback learning task. Following this, a response time adjustment paradigm (the clock task) was conducted, in which subjects had to explore the optimal response time (RT) for reward maximization. The behavioral data from the clock task were then analyzed in relation to feedback-related brain activity from the probabilistic learning task. By this we intended to establish the link between the ability to optimize response speed in the clock task (e.g., optimized reaction time as a subject-specific behavioral parameter) and the neural correlates implicated in mediating the preference for “Go” over “NoGo” learning and vice versa. This procedure is similar to the one employed by Hariri et al. (2006). In this study the behavioral parameter obtained from a delay discounting task conducted outside of the scanner (i.e., the subject-specific delay discounting parameter *k*) correlated with reward-related activity in the ventral striatum assessed via fMRI. In the behavioral delay discounting task, subjects were given the option to choose between a smaller immediate reward and a higher reward that was to be obtained later in time and thus had to be waited for (i.e., the “delayed reward”). The delay discounting task bears some resemblance to the clock task, since it requires an estimation of time combined with an associated reward value. Hence, we chose to follow the approach used by Hariri et al. (2006) in order to investigate if feedback-related neural responses correlated with post-scan response time adjustment parameters.

Based on the above mentioned findings, we predicted preferences for Go over NoGo learning observed in the probabilistic learning task to correlate with the favored learning style in the clock task. Individuals who were prone to learning via positive reinforcement (Go learning) were expected to display a greater ability to adapt their RT to a high speed for reward maximization. In contrast to that, those learning better to avoid punished options were expected to more easily adjust RTs to a slow pace and to wait for a higher reward. As to a possible effect of menstrual cycle, we hypothesized that particularly during the FP, in the presence of high E2 levels, subjects should have difficulties to be patient and to wait for the highest possible reward in a condition that requires slowing down. On the neural level, we further predicted correlations between RT adaptation in the clock task and feedback-related activity in regions of the mesolimbic dopamine system. In particular, the rostral cingulate zone (RCZ) has been shown to be involved in learning from errors and negative feedback (NoGo learning) (e.g., Klein et al., 2007b; Jocham et al., 2009). This led to our assumption that differences in RCZ activity during negative feedback should be predictive of RT adaptations in the clock task across the menstrual cycle, especially when subjects had to wait for a higher reward (i.e., the SLOW condition). Additionally, we expected opposite correlations with RT adaptation in regions that have been associated with reward valuation and positive reinforcement learning such as the ventromedial prefrontal cortex (VMPFC) (see Kringelbach and Rolls, 2004; McClure et al., 2004; Fellows, 2007; Hare et al., 2008; Grabenhorst and Rolls, 2011; Jocham et al., 2011; Peters and Büchel, 2011 for overview).

## Materials and methods

### Subjects

Fourteen healthy female subjects (mean age ± SD: 24.9 ± 1.6; age range: 22–28 years) participated in the fMRI and the behavioral study twice during their menstrual cycle. All subjects were right-handed and had no history of psychiatric, neurological, or hormonal illnesses or other forms of chronic disease (e.g., diabetes). Further exclusion criteria were the use of any medication on a regular basis or of hormonal contraceptives. Subjects were also screened for a regular menstrual cycle with an average cycle length between 26 and 32 days (mean cycle length ± SD: 29.8 ± 2.7).

All subjects gave written informed consent and were paid for participation. This study was approved by the local ethics committee of the medical association of Hamburg (Aerztekammer Hamburg).

### Cycle monitoring and saliva analysis

In this experiment, we applied a counter-balanced within-subject design. Each subject was tested twice during her menstrual cycle: once in the late follicular phase (FP) and a second time in the mid luteal phase (LP). To determine the right time point for the two test sessions, subjects were asked to monitor their cycle with the Clearblue Fertility Monitor® and to inform the experimenters about the displayed details. Test days were then arranged individually according to the course of the subject's cycle. On average (mean ± SD), the follicular test took place on day 13.3 ± 2.3 of a regular cycle, 3.9 ± 1.6 days before the Lutropin (LH) surge triggered ovulation. The luteal test took place on day 24.1 ± 2.0, 7.3 ± 2.8 days after the LH surge and 5.4 ± 2.1 days before the next cycle began.

In addition, morning saliva samples were collected at the 2 days of testing and the day following the onset of menstruation. This was done to ensure that the two sessions were in fact set in the targeted cycle phases by analyzing concentrations of free, bioactive E2 and PROG. A second reason for assessing hormonal parameters was the fact that young women still undergo anovulatory cycles occasionally. Thus, only subjects showing a post-ovulatory increase in PROG in the LP relative to the FP, which is a reliable sign of successful ovulation, were included in the analyses. A 17beta-Estradiol Luminescence Immunoassay and a Progesterone Luminescence Immunoassay purchasable from IBL International were used to determine E2 and PROG concentrations following the standard procedure described in the manual. The sensitivity of the 17beta-Estradiol kit is denoted to be 0.3 pg/mL at the 2 SD confidence limit and for the progesterone kit 2.6 pg/mL.

All 14 subjects showed increased PROG levels in the LP compared to the FP (salivary PROG concentration [mean ± SD]: FP = 51.65 ± 34.97 pg/mL; LP = 141.40 ± 94.41 pg/mL). Conversely, mean E2 levels were higher during FP than during LP (salivary E2 concentration [mean ± SD]: FP = 4.56 ± 3.84 pg/mL; LP = 4.02 ± 2.59 pg/mL).

### The clock task

**Figure 1:**
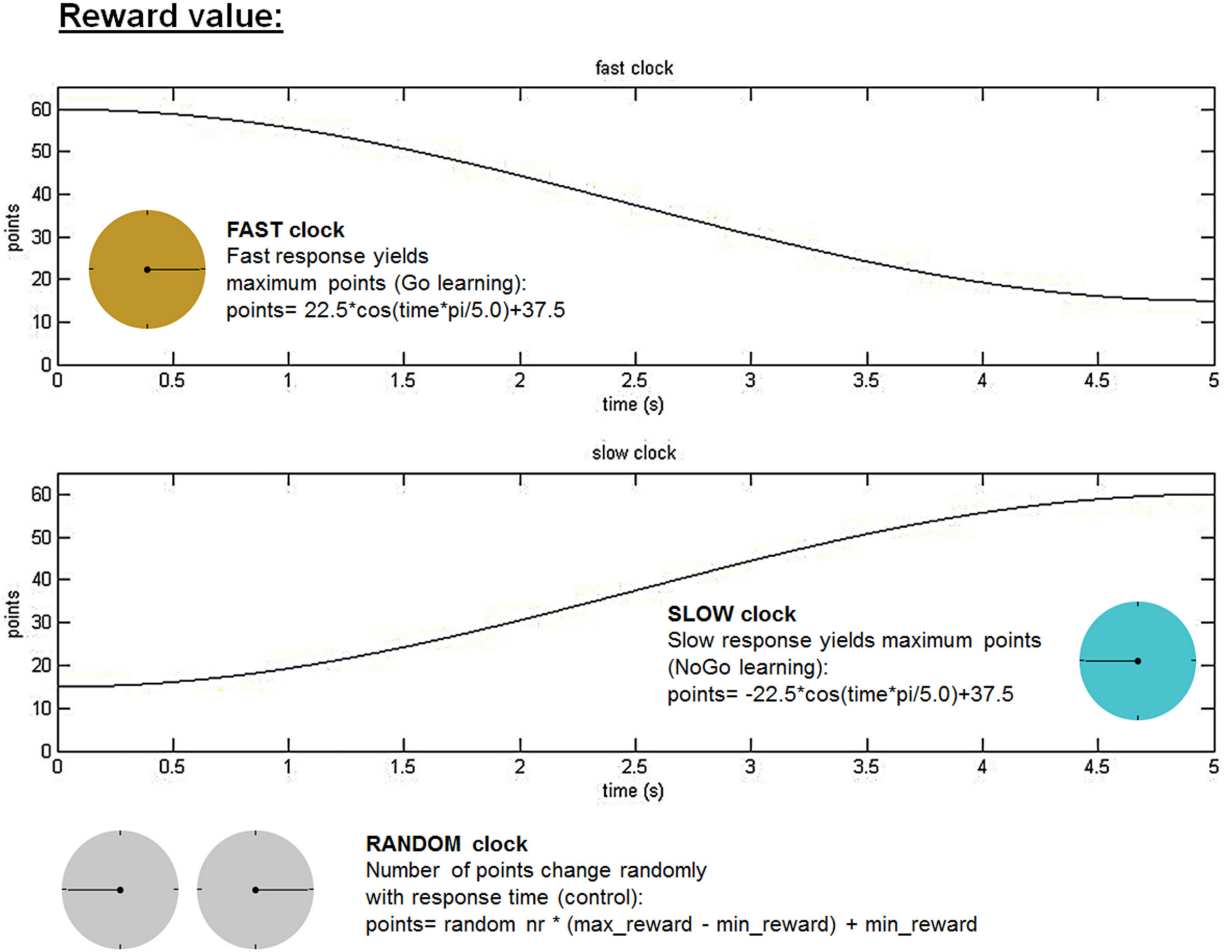
**Clock task**. Reward values varied as a cosine function of response time. Subjects were instructed to figure out the optimal time point during a whole clock-arm turn of 5 s to stop the ticking clock and achieve maximum reward. In the fast clock condition, fast responses yielded the highest reward value (FAST clock for “Go learning”), whereas in the slow clock subjects had to wait longer to win maximum points (SLOW clock for “NoGo learning”). The RANDOM clock acted as a control condition, in which there existed no relation between response time and reward value. In all clock conditions random noise ranging between −5 and 4 points was added to the reward value in order to prevent subjects from memorizing an exact reward value at a specific response time. The three different clock types were presented in three separate runs for each clock consisting of 50 trials, which were counterbalanced across subjects and cycle phases.

Subjects performed a response time adjustment paradigm (modified from Moustafa et al., 2008) outside of the scanner, which we called the “clock task.” In this task, subjects were presented with three different running clock faces indicated by different colors, and were instructed to explore at which point in time they had to press a stop button in order to maximize personal reward. Reward value, as a function of response time (RT) during a full clock-arm turn of 5 s, varied between the different clocks. For one clock fast responses yielded highest reward (FAST clock favoring Go learning). In contrast, a second clock required patience, meaning that subjects had to delay responding to maximize reward (SLOW clock favoring NoGo learning). The exact reward value of each trial in the FAST and the SLOW clock condition was calculated with a cosine function, but always ranged between a minimum of 15 and a maximum of 60 (see Figure 1). The third clock acted as a control condition with no contingency between response time and magnitude of reward outcome (RANDOM clock, in which points varied randomly across given response times). Exact reward values in the RANDOM condition were determined by multiplying a random number with the difference between the maximum and minimum reward value (i.e., 60–15) and adding the minimum reward value to this product. In all three clock conditions, a random noise parameter (range: −5 to +4 points) was added to the calculated reward value in order to prevent subjects to associate a specific RT with a certain reward value. After each response immediate reward feedback was provided. Feedback was always followed by a blank screen that lasted the residual time that would have been necessary to finish the whole clock arm turn before the next trial began. If no response was given within a full clock arm turn (after 5 s) no points were won and subjects had to wait a period of another 5 s before the next clock face was shown. Each of the three clock types (FAST, SLOW, and RANDOM) was presented 50 times in a row. Between these runs (i.e., 50 trials of one clock type) there was a short break. During this break subjects were informed that they would be confronted with a new type of clock in the following run and were again instructed to figure out the optimal response time. The new run was started as soon as the subject indicated to be prepared. The sequence of the runs for the three clock types was counterbalanced across participants and cycle phases.

### Probabilistic learning task

Subjects underwent fMRI while performing a reinforcement learning task. This task was adapted from previous studies showing that performance in this learning task depends on dopaminergic transmission (Frank et al., 2004; Klein et al., 2007b). Subjects were presented with three different pairs of arbitrary symbols and had to learn which symbol was the most rewarded option. For that, they received probabilistic feedback in form of a smiley or a grumpy after each choice. The three symbol pairs—called “AB,” “CD,” and “EF”—varied in their probability for positive or negative feedback: in the stimulus pair “AB” a choice of “A” yielded positive feedback in 80% of the cases and led to negative feedback in 20%. Choosing “B,” on the other hand, led to a rewarding smiley in only 20% of the cases and to negative feedback with a probability of 80%. The other symbol pairs yielded positive or negative feedback in a corresponding probabilistic manner with a choice of “C” of the pair “CD” being rewarded in 70% and “D” in 30% of the cases. A choice of “E” of the pair “EF” yielded positive feedback with a probability of 60%, whereas “F” was rewarded in 40% of the cases. Thus, in the course of this task, subjects should have either learned that choosing “A” was the most rewarded option or that choosing “B” was the most punished option, depending on their preferences for Go or NoGo learning. To test for learning success, subjects had to complete a second session outside the scanner, in which the symbols were mixed in new pairs (“AC”, “AD”, “AE”, “AF”, “BC”, “BD”, “BE”, “BF”, “CD”, “CE”, “CF”, “DE”, “DF”). Individuals who learned better via reward (Go learning) should display a higher tendency to choose the symbol “A” from these new stimulus pairs, whereas individuals that learned better via punishment (NoGo learning) should tend to avoid “B” in the pairs including the symbol “B.” For further details regarding this task please also see Supplementary Figure S1.

### Behavioral data analyses

SPSS 19 was applied to analyze behavioral data. Prior to statistical analyses, mean RTs for each clock condition were calculated per subject. For that, RTs of less than 150 ms were discarded, since they were presumably caused unintendedly. Additionally, mean RTs of the first 12 trials (i.e., “first block”) and the last 12 trials (i.e., “last block”) of the three clock conditions were calculated for each subject (see Moustafa et al., 2008 for a similar procedure). Mean RTs of the first block allowed us to investigate the initial response speed indicating the subject's tendency to respond prior to learning in a state of uncertainty. Mean RTs of the last block indicated the optimized response speed near to the end of each clock condition, which represents a subject's best learning outcome. To obtain measures of Go and NoGo learning, relative response speed was determined. This was done by subtracting mean RTs of the FAST clock condition from mean RTs of the RANDOM condition (i.e., “relative speeding”), as well as subtracting mean RANDOM clock RTs from the mean SLOW clock RTs (i.e., “relative slowing”). This was also done for first and last block RTs, relative to first and last block RTs of the RANDOM clock, respectively. Since we were most interested in the time adjustment, i.e., the behavioral adaptation, over the course of each clock condition, differences between mean RTs of the last and the first block were calculated. Pearson correlations were performed to test for an association between RTs of the clock task and learning performance during the probabilistic learning task. A 2 × 2 repeated-measures ANOVA including the factors clock type (FAST, SLOW) and cycle phase (FP, LP) was computed to test for a possible effect on relative response time. Time adjustment and thus learning outcome, represented by the RT change from the first to the last block, was compared between the two cycle phases for both clock types using paired *t*-tests. Since we had clear a priori hypotheses regarding the direction of associations or differences in Go vs. NoGo learning in the two cycle phases, we report one-tailed significances if not otherwise indicated.

### fMRI data acquisition and analyses

The learning part of the probabilistic learning task, in which subjects were presented with positive or negative feedback according to their choices, was performed while brain activation was measured with fMRI. Imaging was conducted on a 3 T MRI scanner (Siemens TRIO). Thirty-three axial slices were acquired parallel to the anterior commissure – posterior commissure plane (voxel size = 2 × 2 × 2 mm^3^, distance factor = 50%, descending direction) covering the whole brain. Functional images were obtained using a gradient echo planar imaging (EPI) sequence [repetition time (TR) = 2000 ms, echo time (TE) = 25 ms, field of view = 216 mm] resulting in a total of 738 image volumes. A high-resolution structural scan was acquired for each subject using a three-dimensional magnetization-prepared rapid-acquisition gradient echo sequence (MPRAGE) following the functional scans. Subjects viewed the experiment on the display through a mirror mounted on the head-coil and gave manual responses with a five-button keypad. Preprocessing and statistical analyses of functional data were performed using SPM8 (Wellcome Department of Cognitive Neurology, University College London, London, UK). Preprocessing steps included coregistration to the anatomical image, correction for motion artifacts (realignment and unwarping), for acquisition time differences (slice-timing), and for low-frequency fluctuations, normalization into standard stereotactic space (EPI template by the Montreal Neurological Institute, MNI), and spatial smoothing using a 6 mm full-width half-maximum isotropic Gaussian kernel. A general linear model (GLM) was defined to obtain parameter estimates of event-related activity in each voxel for each subject in both cycle phases. Positive and negative feedback (at time of feedback onset) were modeled as independent regressors convolved with the canonical hemodynamic response function (hrf). Linear *t* contrasts against implicit baseline were defined to explore the specific effects of each feedback type. The resulting contrast images were then passed to the second level analyses, in which correlations between feedback-related activity and RT adjustment in the clock task were assessed for both cycle phases using regression analyses.

All reported activations were significant at a voxel-level threshold of *p* < 0.001 uncorrected and survived a cluster-level family-wise error (FWE) correction for multiple comparisons with *p* < 0.05. For our a priori regions of interest, namely the RCZ and the VMPFC, we used small volume corrections (SVC, Worsley et al., 1996) and applied spherical volumes (radius = 10 mm) at the activation maxima reported by Klein et al. (2007b) for the RCZ (MNI-coordinates: *x* = 6, *y* = 30, *z* = 29) and by McClure et al. (2004) for the VMPFC (MNI-coordinates: *x* = −8, *y* = 48, *z* = −4). The coordinates by Klein et al. (2007b) were originally reported in Talairach space and thus converted to MNI reference space with GingerALE 2.1.1 using the Lancaster transformation (Lancaster et al., 2007). Activations corrected for small volume are reported at a voxel-level threshold of *p* < 0.05, corrected. Parameter estimates from the local activation maximum in the regions found to be associated with performance in the clock task were extracted with marsbar (available at: http://marsbar.sourceforge.net).

## Results

### Behavioral results

**Figure 2:**
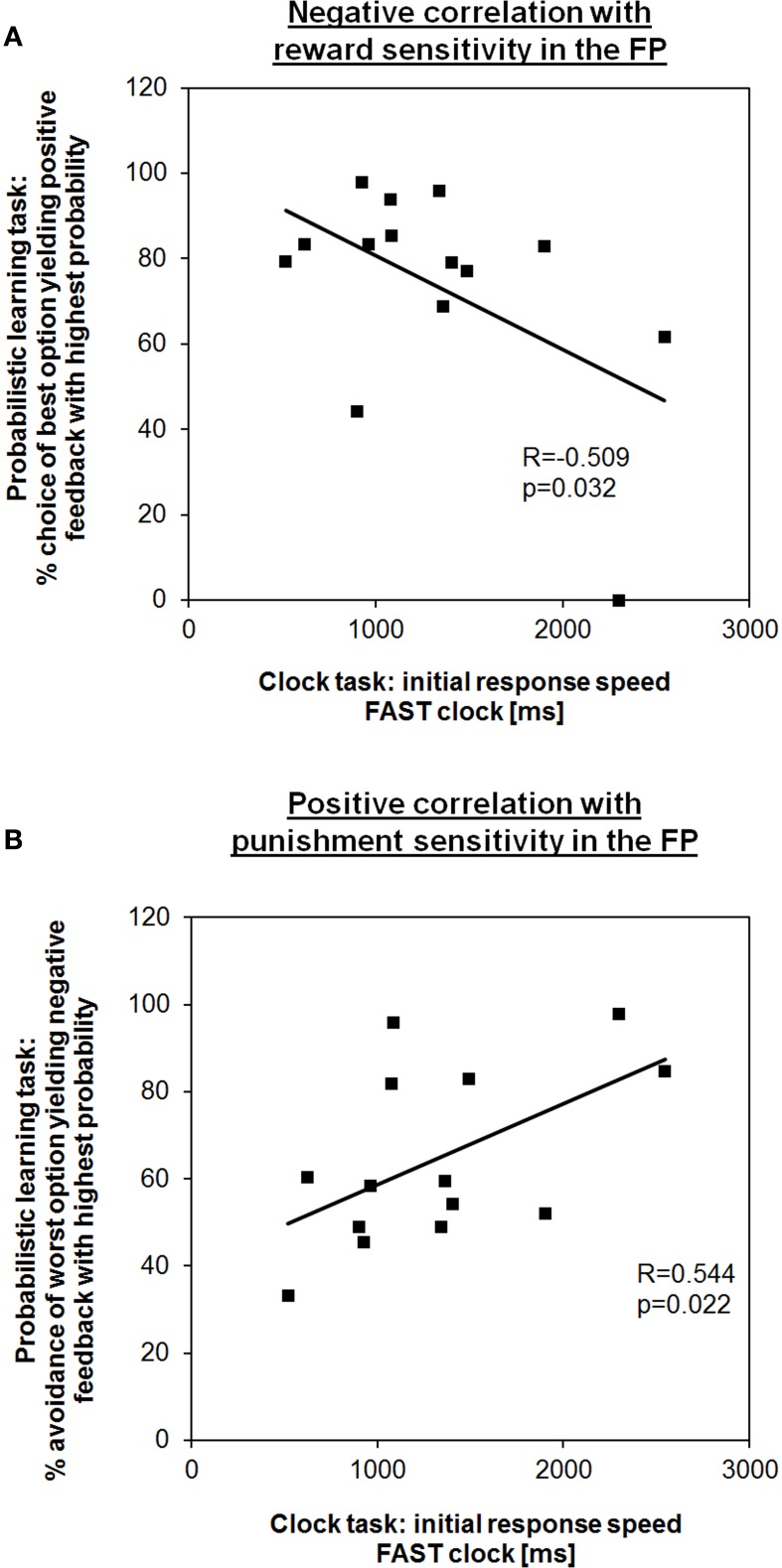
**Learning outcome from the probabilistic learning task predicts response speed in the first block of the fast clock condition during the FP. (A)** Subjects who showed a higher percentage of choosing the best option in the second test session of the probabilistic learning task (i.e., “Go learners”) were also better able to speed up during the fast condition at the very beginning. **(B)** In contrast, subjects who showed a higher tendency to avoid the most punished option (i.e., “NoGo learners”) took longer to respond.

First, we tested for coherence between time adjustment in the clock task and learning outcome in the probabilistic learning task. In line with our hypothesis, in the FP the initial response speed in the FAST clock condition (i.e., mean RT of the first block) showed a negative correlation with the frequency of choosing the best option (i.e., the letter “A”) from new letter pairs in session 2 of the probabilistic learning task (*R* = −0.509, *p* = 0.032; Figure 2A). Hence, subjects who learned better via positive feedback were also responding faster at the beginning indicating that a preference for speeding up is also a form of “Go learning.” RT adaptation in the FAST clock condition might be more intuitive for female subjects during the FP than during the LP, in which no such correlation could be observed (*R* = 0.324, *p* = 0.129). Also consistent with our predictions, during the FP the initial RT in the FAST clock was positively associated with the percentage of successful avoidance of the worst option (i.e., the letter “B”) in new letter pairs (*R* = 0.544, *p* = 0.022; Figure 2B). According to this, a relative preference for avoidance learning was associated with a slower initial response tendency. Again, there was no equivalent correlation found for the LP (*R* = −0.034, *p* = 0.454). This further adds to the assumption that during the FP enhanced RTs in the FAST clock condition may be facilitated by a propensity for Go learning, while at the same time the ability to learn from punishment (NoGo learning) was compromised. Regarding the SLOW clock no correlations between initial response speed and performance in the learning task could be found. Also, there were no correlations between initial response speed and reinforcement learning during the LP. The observed associations in the FAST clock condition are in line with previous findings showing that the clock task is applicable to test for dopamine modulated learning performance in the sense of response time (Moustafa et al., 2008).

**Figure 3:**
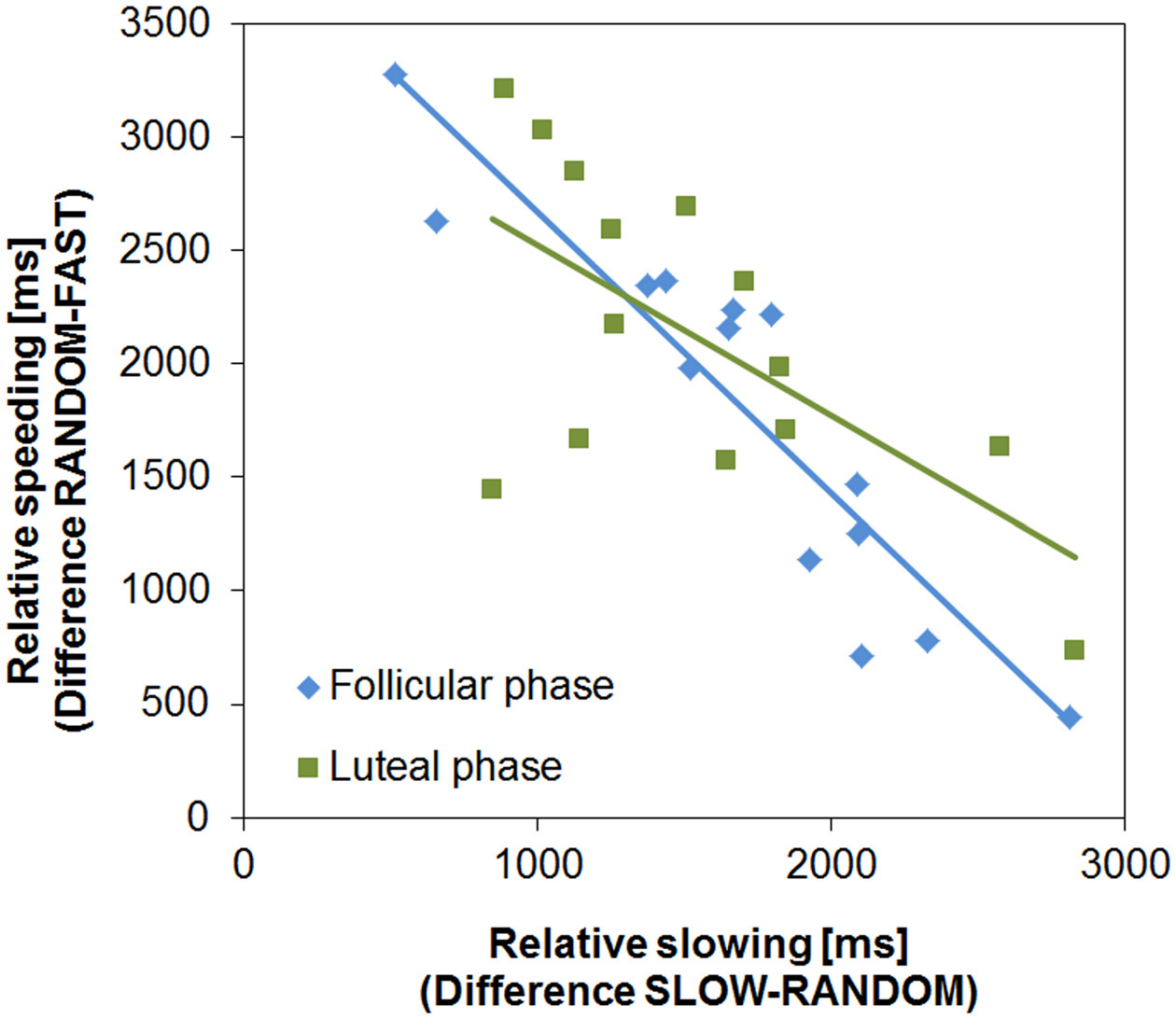
**Functional opponency of slowing and speeding**. Relative slowing and speeding were negatively correlated in both cycle phases (FP: *R* = −0.918, *p* < 0.001; LP: *R* = −0.637, *p* = 0.014).

Next, we examined if the two learning processes, relative speeding (Go) and slowing (NoGo, for a similar procedure see Moustafa et al., 2008), were associated. As predicted, relative speeding and slowing were negatively correlated in both cycle phases (FP: *R* = −0.918, *p* < 0.001; LP: *R* = −0.637, *p* = 0.007; Figure 3). In the FP this association was slightly stronger than in the LP, which was indicated by the almost significant difference between the two correlations (Fisher's *z* = −1.93, *p* = 0.053, two-tailed). Subjects more prone to Go learning were good at speeding up during the FAST condition, while they had difficulties in slowing down (i.e., NoGo learning) in the SLOW condition. Again, this points to the functional opponency of the two learning processes and supports the view that the clock task is suitable to assess the cycle dependent modulation of temporal decision making.

Finally, we tested for an effect of menstrual cycle phase on RT adjustment and preferences for Go over NoGo learning and vice versa. Subjects showed a similar baseline response speed in both cycle phases with mean RTs in the RANDOM condition being equivalent in the FP and LP (*T* = 1.035, *df* = 13, *p* = 0.320, two-tailed; Mean RT RANDOM clock [mean ± sem]: FP = 2433 ± 199 ms, LP = 2146 ± 143 ms). To test for an effect of cycle phase on RT in the two clock types a 2 (clock type: FAST vs. SLOW) × 2 (cycle phase: FP vs. LP) repeated-measures ANOVA was run with the mean RTs. There was a main effect of clock type [*F*_(1, 13)_ = 1119.01, *p* < 0.001] in that RTs in the SLOW clock condition were generally higher than in the FAST clock (*T* = 33.45, *df* = 13, *p* < 0.001; Mean RTs [mean ± sem]: SLOW = 4243 ± 74 ms, FAST = 669 ± 54 ms,). However, there was no significant interaction between clock type and cycle phase in the different clock conditions for the RTs averaged over the complete run of 50 trials. The same applied to mean RTs of the last block of trials (i.e., optimized response speed). Solely, when considering the initial response speed during the first block of trials, a significant interaction between clock type and cycle phase could be found [*F*_(1, 13)_ = 6.020, *p* = 0.029]. *Post hoc t*-Tests revealed that in the SLOW clock condition subjects initially reacted more appropriately during the LP than during the FP (*T* = −2.048, *df* = 13, *p* = 0.031; initial RT SLOW clock [mean ± sem]: LP = 3574 ± 230 ms, FP = 3273 ± 190 ms).

The more exact measures of Go and NoGo learning in form of RT adjustment in relation to baseline RT in the RANDOM clock (i.e., relative speeding and relative slowing) revealed no interaction between cycle phase or clock type either. Nonetheless, the mean relative RTs from the last block of trials correspond numerically to the expected pattern (FAST condition [mean ± sem]: FP = 2183 ± 343 ms, LP = 1660 ± 251 ms; SLOW condition: FP = 1925 ± 356 ms, LP = 2456 ± 262 ms) although differences failed to reach statistical significance [clock type × cycle phase: *F*_(1, 13)_ = 0.828, *p* = 0.379]. Note that for the relative RTs a high value indicated better RT adjustment to the respective clock type.

**Figure 4:**
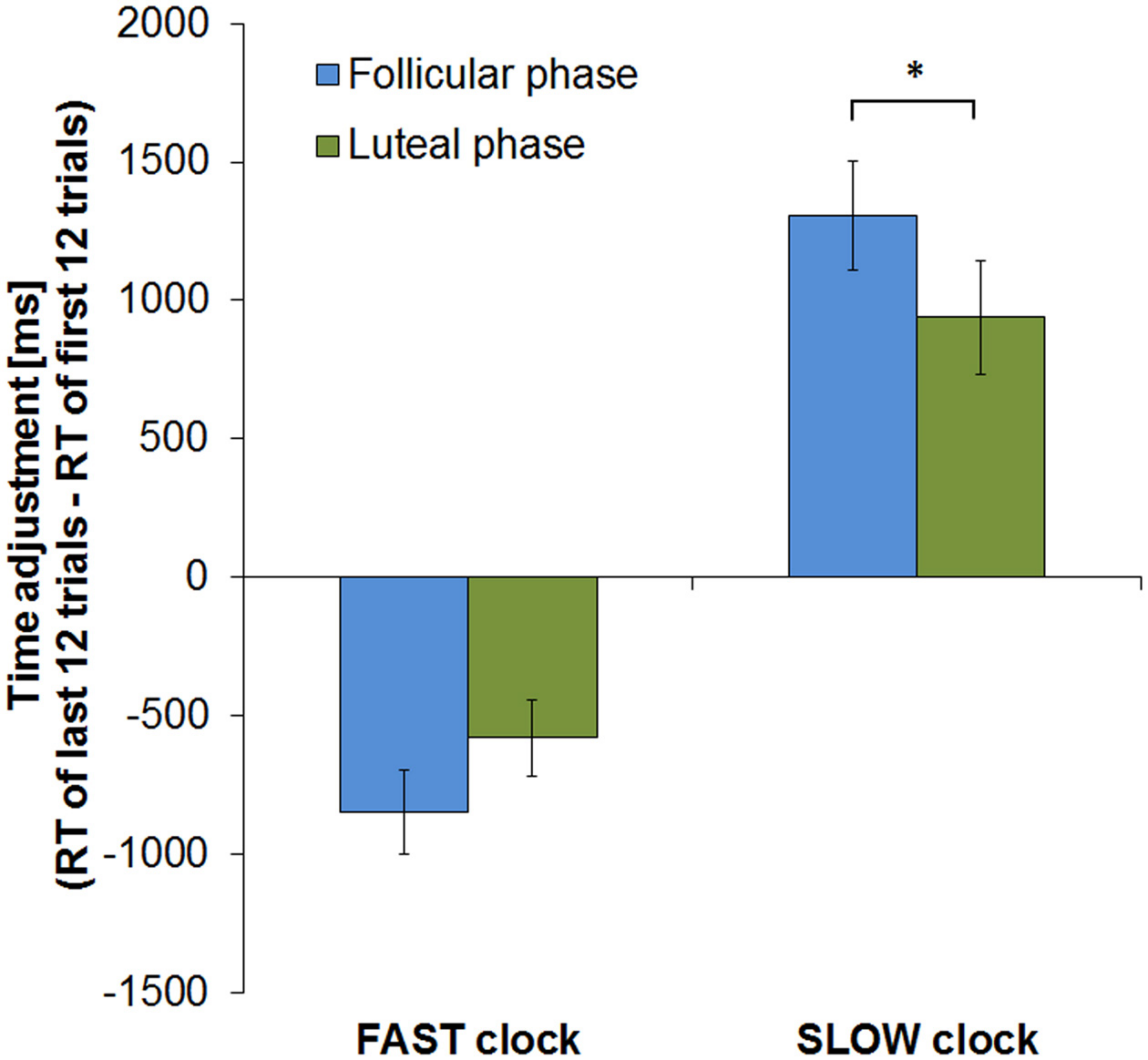
**RT change from first to last block of the clock task in both cycle phases**. In both clock conditions subjects showed greater RT change in the course of the experimental run during the FP (^*^p < 0.05). Error bars represent standard error of the mean (sem).

Considering the course of learning by comparing time adjustment from the beginning to the end of the task (calculated by subtracting mean RT of the first 12 trials from those of the last 12 trials) revealed, however, a significant interaction between cycle phase and clock type [*F*_(1, 13)_ = 5.707, *p* = 0.033]. During the FP subjects displayed a higher level of response time adjustment in the SLOW clock condition by showing a greater RT change from the first to the last round than during the LP (*T* = 2.412, *df* = 13, *p* = 0.016; RT change [mean ± sem]: FP = 1304 ± 198 ms, LP = 939 ± 204 ms). In the FAST clock condition, mean RT changes in the two cycle phases did not significantly differ between cycle phases (*T* = −1.435, *df* = 13, *p* = 0.088; RT change [mean ± sem]: FP = −849 ± 152 ms, LP = −514 ± 152 ms). The fact that the difference in RT change between the FP and LP was only significant in the SLOW clock condition task, but not in the FAST, suggests that slowing down puts more challenge on subjects during the FP than speeding up. Slow reponses might therefore require a higher level of adaptation due to the higher need for compensation during the FP. Hence, follicular subjects displayed an even greater RT change during the SLOW clock condition than during the FAST. Figure 4 shows the RT changes of both cycle phases in the two clock conditions.

### Imaging results

To test for a link between response time adjustment in the clock paradigm and feedback-related activity in the probabilistic learning task, regression analyses with mean RTs of the different clock conditions and the neural responses based on the contrasts “negative feedback > baseline” and “positive feedback > baseline” were performed.

#### Follicular phase

**Figure 5:**
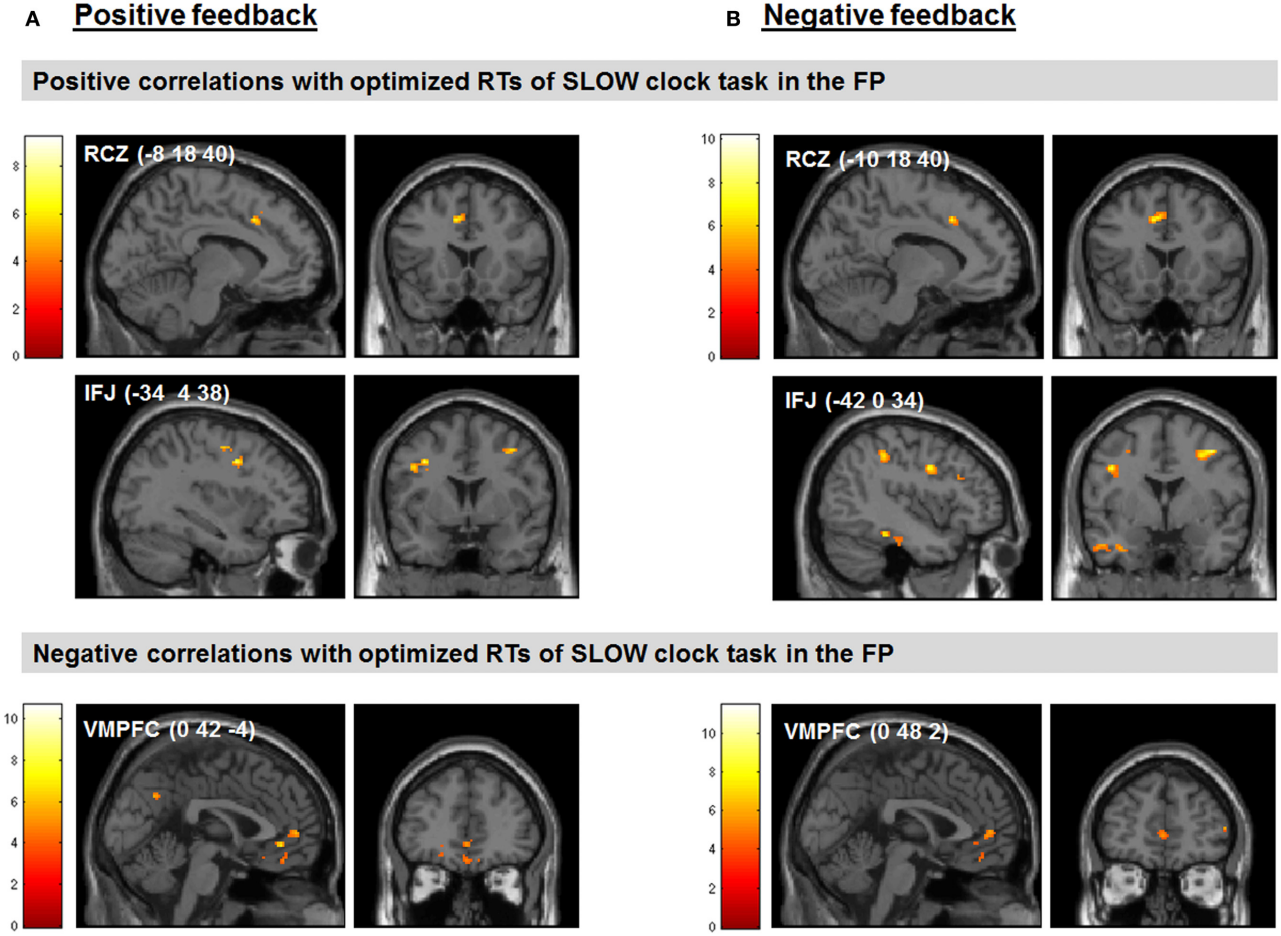
**Feedback-related activity during probabilistic learning predicted performance in the last block of the SLOW clock during the FP. (A)** Correlations between neural responses based on the contrast positive feedback > baseline in the probabilistic learning task and optimized RTs of the last block during the SLOW clock condition. **(B)** Correlations between neural responses based on the contrast negative feedback > baseline in the probabilistic learning task and optimized RTs of the last block during the SLOW clock condition. Displayed positive correlations survived FWE correction for multiple comparisons at cluster-level (*p* < 0.05). The negative correlations are reported at *p* < 0.05, SVC (based on the coordinates reported in McClure et al., 2004).

We found several correlations between the optimized final response speed in the SLOW clock condition (i.e., mean RT of the last block) and feedback-related activity during probabilistic learning. Note that the SLOW clock condition presumably represented the hardest challenge for subjects during the FP. Regarding our a priori regions of interest, increased activation in the RCZ (*x* = −8, *y* = 18, *z* = 40) and reduced activity in the VMPFC (*x* = 0, *y* = 42, *z* = −4) in response to positive feedback as compared to baseline were predictive of slower RTs in the last block of the SLOW clock condition. Furthermore, signal increases in the left IFJ (*x* = −34, *y* = 4, *z* = 38) in response to positive feedback were positively associated with RTs in the SLOW clock condition (see Figure 5A for clusters found to be associated with effective RT adaptation based on the contrast “positive feedback > baseline”).

**Figure 6:**
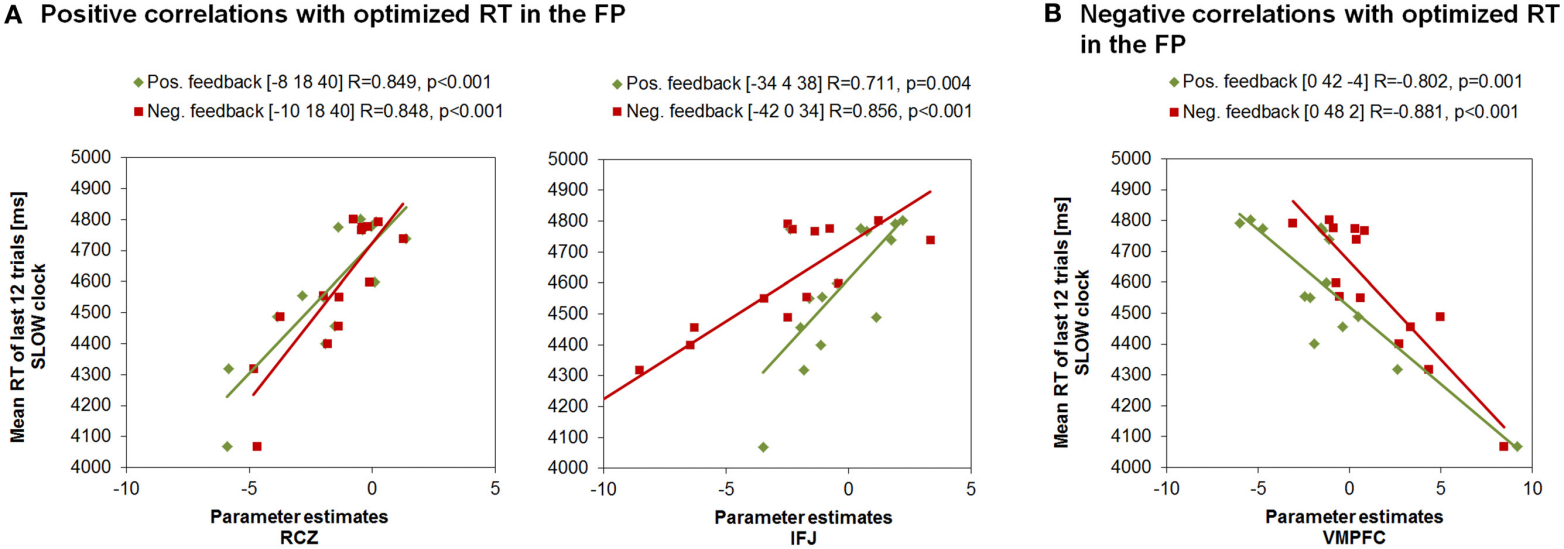
**Correlations between RTs of the last block in the SLOW clock and the parameter estimates of peak voxels in feedback-related clusters found to be associated with response time adjustment in the SLOW clock during FP. (A)** Increased activation in RCZ and IFJ predicted effective response time adaption in the SLOW clock. **(B)** Increased activation in the VMPFC was associated with compromised RT adaptation in the SLOW clock.

Interestingly, the same neural correlates of optimal RT adaptation in the SLOW clock were found in the “negative feedback > baseline” contrast. A slower optimized response speed could be predicted by a signal increase in the RCZ (*x* = −10, *y* = 18, *z* = 40), whereas a poorer RT adaptation toward the end of the SLOW clock condition was accompanied by increased activation in the VMPFC (*x* = 0, *y* = 48, *z* = 2). In addition, stronger responses in the left IFJ (*x* = −42, *y* = 0, *z* = 34) were predictive of slower final RTs (Figure 5B). Furthermore, during both feedback types optimized RTs in the SLOW clock correlated positively with activations in the precentral sulcus and the inferior temporal lobe and negatively with activation in the posterior superior temporal sulcus (post STS) and the middle occipital cortex (MOC) (Table 1 lists all activations that were found to be associated with optimized response speed in the SLOW clock condition during the FP). The correlations between optimized RT in the SLOW clock condition and the parameter estimates extracted from the local activation maxima of the clusters in the RCZ, IFJ, and VMPFC further point to the resemblance of the brain-behavior correlations in response to positive and negative feedback and indicate that these results were not driven by outliers (Figure 6). No correlations were found between feedback-related brain activity and RTs in the FAST clock condition.

**Table 1:** **Brain activations in response to negative and positive feedback during probabilistic learning which correlated with optimized RTs (last block) of the SLOW clock condition in the follicular phase**.

**Region**	**Neural activations in response to positive feedback (positive feedback > baseline contrast) associated with last block RTs of the SLOW clock condition**					**Neural activations in response to negative feedback (negative feedback > baseline contrast) associated with last block RTs of the SLOW clock condition**				
	***x***	***y***	***z***	***T***	**Cluster size**	***x***	***y***	***z***	***T***	**Cluster size**
**POSITIVE CORRELATIONS**										
RCZ	−8	18	40	6.44	77	−10	18	40	7.20	126
L IFJ	−34	4	38	7.29	48	−42	0	34	7.47	99
R SFS/precentral sulcus	38	0	48	6.06^b^	68	38	0	48	8.15	112
L Inferior temporal lobe	−44	−36	−18	9.24	142	−44	−36	−18	10.15	208
**NEGATIVE CORRELATIONS**										
VMPFC	0	42	−4	5.041^a^	4	0	48	2	5.36^a^	13
R Posterior STS/MTG	48	−52	24	10.65	177	52	−52	18	11.45	139
MOC	−44	−76	34	8.23	116	−42	−76	34	5.45	92

Activations are reported at p_FWE_ < 0.05 (cluster-level) if not otherwise indicated.

a p<0.05, SVC.

b p<0.001, uncorrected (voxel-level).

#### Luteal phase

Feedback-related activity in the LP was not associated with RT adaptation in any of the three clock types. This corresponds to the lack of correlations between RT adaptation and the behavioral data of the probabilistic learning task in the LP.

## Discussion

In this study we investigated whether naturally occurring variations in E2 and PROG levels influence the ability to adapt response speed in the sense of Go vs. NoGo learning (i.e., speeding up vs. slowing down) over the course of the menstrual cycle. Indeed, we observed that in the FP as compared to the LP subjects displayed a higher need for adaptation of their response speed in the condition that required slower responses for reward maximization. The rather compromised ability to adapt to a slow response speed during the FP was also reflected in the observed correlations between optimized response speed in the last block of the SLOW clock condition and follicular brain activation in response to positive and negative feedback during a probabilistic learning task. While increased activation of the RCZ and the IFJ was associated with better RT adaptation to a slow speed, strong responses in the VMPFC predicted poorer performance in this condition.

In sum, our results indicate an effect of menstrual cycle phase on NoGo learning processes in the temporal domain. These findings support our hypothesis that increased E2 levels during the FP may lead to a Go learning bias and an impaired ability to learn via the NoGo pathway. Against the background of growing evidence from rodents that describes an opposing effect of E2 and PROG on striatal DA levels, in that E2 enhances whilst PROG acts inhibiting on dopaminergic tone (Luine and Rhodes, 1983; Dluzen and Ramirez, 1984; Majewska et al., 1986; Mauvais-Jarvis et al., 1986; Lévesque et al., 1989; Becker, 1999), our data are consistent with previous studies reporting an effect of striatal DA on action selection (Frank et al., 2004; Klein et al., 2007b; Moustafa et al., 2008). All these previous results on the association between DA and action selection had been derived from testing individuals with differences in dopaminergic functioning (i.e., Parkinson patients on and off medication or carriers of specific genetic polymorphisms concerning D2 receptor density). Collectively, they point to an increased preference for Go over NoGo learning in a state of high as compared to low dopaminergic tone. This is in line with our observation of an impaired learning ability in the SLOW condition during the FP. In that phase high levels of E2 are unopposed by PROG and are presumed to increase DA levels, which is why they should indirectly cause a Go bias. In the present study, subjects in the FP displayed a higher need for RT adaptation in the SLOW clock condition, which acted as a measure of NoGo learning performance. Considering the greater change of RT over the course of the SLOW clock during the FP, our results suggest that subjects were able to compensate for their impaired ability to adapt to a slow pace, by taking more effort in response speed adaptation. However, this adaptation may not have been as sufficient as the performance in the LP, which was indicated by slightly poorer relative slowing of follicular subjects. The fact that relative speeding and slowing did not significantly differ between the two cycle phases potentially indicates that effects of naturally occurring hormonal shifts during the menstrual cycle are not necessarily very strong. As a result, they may rather be compensated without greater effort in an explicit behavioral task such as the clock task. Altogether, our data support the theoretical model of a DA dependent learning style (e.g., Frank et al., 2004; Cools, 2008) and propose natural variations of the steroid hormones E2 and PROG as other important factors that need to be considered. Apart from its biasing effect toward the Go pathway in reward-based learning, E2 has also been found to increase the internal clock speed (Sandstrom, 2007; Pleil et al., 2011). In our study, subjects in the FP might therefore have overestimated the passing time and given their response relatively sooner. This explanation could also account for the initially faster and thus less suitable response speed in the SLOW clock condition that was observed during the FP. Again, this accelerating effect of E2 is presumably driven by an interaction with the DA system. DA has been found to be involved in time perception by studies reporting an increase of the internal clock speed in response to DA agonists (e.g., Meck et al., 1996). Hence, in the presence of high E2 levels one may speculate that heightened DA levels might have also promoted an acceleration of internal clock speed. This assumption also seems plausible when considering that both enhanced DA levels as well as heightened E2 may increase individual delay discounting tendencies in man and animals (Winstanley, 2011; Smith et al., 2014), which in part depend on the subjective feeling of passing time as well.

As to the translation of adjusting response time to a fast vs. a slow speed to Go vs. NoGo learning, we argue that these two learning styles, which are usually assessed by reward-based learning tasks (i.e., reinforcement learning), are also involved in response time adjustment. The fact that we applied both types of tasks, the clock task for time adjustment and the probabilistic learning task for reward-based learning, allowed us to examine if performance in the two paradigms both rely on Go and NoGo learning processes and how the two tasks are associated with each other. We found correlations between the initial response speed in the FAST clock condition and the learning outcome in the probabilistic learning task during the FP that support the view that RT adjustment to a fast vs. a slow speed is equivalent to Go vs. NoGo learning processes (Moustafa et al., 2008). Specifically, subjects who were better in speeding up also showed higher reward sensitivity in the probabilistic learning task, whereas their avoidance of the punished option was less successful. Thus, the Go learning condition of the clock task (i.e., the FAST clock) was positively associated with Go learning as well as negatively associated with NoGo learning in feedback learning.

On the neural level our results indicate antagonistically acting brain-behavior correlations. We found that in the FP increased activity in the RCZ was predictive of a successful RT adaptation toward the end of the SLOW clock condition. Since the RCZ plays an important role in NoGo learning, as indicated by its frequent observation during learning from errors and negative feedback (Fiehler et al., 2004; Klein et al., 2007a; Jocham et al., 2009), an increased activity in this region could have helped subjects to perform better in a task that required NoGo learning in the form of waiting longer for a higher reward. Klein et al. (2007b) applied a reinforcement learning task resembling our version of the probabilistic learning task and compared two groups differing in their genetically determined D2 receptor density. The group with a high receptor density showed an increased signal in the RCZ in response to negative feedback and this group was also better at avoiding a negative outcome in the behavioral post-test. The D2 receptor type has been proposed to be implemented in the NoGo pathway (Frank et al., 2004) and therefore an increased receptor density of this type should cause a bias toward NoGo learning. Since the high E2 levels are thought to downregulate D2 receptor density and may decrease receptor binding (Bazzett and Becker, 1994; Becker, 1999), individuals that show higher sensitivity of the RCZ to NoGo signals in form of negative feedback might be better adapted to succeed in the SLOW condition of the clock task. The cluster in the RCZ found in this study did not survive small volume correction based on the previously published coordinates by Klein et al. (2007b), which is why our result considering the RCZ may be interpreted cautiously. It might be that small location differences between the RCZ cluster reported here and that of Klein et al. (2007b) are due to fact that we only tested female subjects while the other study investigated men. In fact, sex differences in brain volume (e.g., Cosgrove et al., 2007) may account for slight locational drifts to some extent. Nonetheless, the anatomical location of the cluster reported here corresponds to the literature about the RCZ (see for example Ridderinkhof et al., 2004). Thus, our results further underline the importance of the RCZ for NoGo learning by pointing out that in the FP subjects may have benefited from a stronger RCZ response during feedback learning, which also led to an advantage in the SLOW clock condition.

Similarly, in the FP subjects with heightened activity in the IFJ during feedback processing were more successful to adapt in the SLOW clock. Since the IFJ has been consistently implicated in cognitive control (Brass et al., 2005; Derrfuss et al., 2005), increased activity in this region might have helped subjects in the FP to perform better in this counterintuitive task, in which they had to counteract the tendency for immediate responding. In contrast, decreased activation in the VMPFC was associated with better RT adaption to a slow response speed. This is in line with observations that increased activation in this brain region is associated with preferences for immediate reward and may promote delay discounting tendencies both in the context of longer time scales (McClure et al., 2004) as well as in the range of seconds (McClure et al., 2007). In addition, the VMPFC may also play a role in the representation of relative reward value und reward-related preferences (e.g., Grabenhorst and Rolls, 2011). In the context of the probabilistic learning task this region has been found to show an increased activity in the post-learning test phase in response to Go trials, in which subjects should select the most rewarded option (i.e., “AC,” “AD,” “AE,” “AF”) (Jocham et al., 2011). More importantly, this effect was found in subjects having received a dose of amisulpride, a D2 antagonist, but it was absent when the same subjects received placebo. Considering this, the VMPFC might be especially implicated in value-based decision making that involves Go learning processes. Therefore, a heightened activity in this region during feedback processing may potentially explain subjects' poor performance in the NoGo condition of the clock task, since a strong response in this brain region could be interfering with a task that requires the NoGo pathway.

In sum, the brain-behavior correlations suggest that effective RT adaption to a slow speed during the FP may be related to enhanced top-down processing involving increased activation in regions implemented in cognitive control and monitoring of behavioral outcomes. On the other hand, enhanced bottom-up processing in areas implicated in reward valuation might be associated with compromised subsequent RT adaptation. The fact that these correlations were only found in the last block of the SLOW clock condition may reflect a subject's maximum individual capacity to compensate for her compromised ability to be patient and to finally withhold the initial urge to respond rather rapidly during the FP. Correspondingly, we found no consistent correlations during the LP in the absence of this need for compensation. The FAST clock condition in turn should also not require much compensation in subjects during the FP, which might explain the lack of equivalent correlations between RT adaptation and brain activity during feedback learning in this condition. This absence of neural correlates predicting the ability to speed up response speed in the Go condition of the clock task corresponds well to the findings of Klein et al. (2007b) who reported mainly differential activation associated with negative feedback (i.e., NoGo learning) when comparing subjects with different D2 receptor densities. Interestingly, in the present study effective RT adaptation could be predicted by neural responses from the same regions independent of feedback-type (see Table 1). It might be that the successful compensation for a compromised capacity for NoGo learning in the FP required a general increase of performance monitoring resulting in an enhanced recruitment of RCZ and IFJ during feedback-processing *per se*.

Important to note, our results indicate neural correlates predicting the ability to adapt response speed on the interindividual level. Although we can only speculate that this may have been caused by possible interactions with other factors we did not include in our design. First, the time of the day on which the tests were carried out could not be controlled across participants. There is, however, some evidence that points to an interaction between time perception and circadian rhythm (e.g., Lustig and Meck, 2001; Kuriyama et al., 2003; Bussi et al., 2014). Also we did not account for possible effects by other hormones. The steroid hormone cortisol has been shown to influence delay discounting (Takahashi, 2004) and might therefore also play a role in time adjustment. Furthermore, genetically determined differences in dopaminergic functioning could have affected our results. For instance, Jacobs and D'Esposito (2011) tested working memory performance of women during different phases of their menstrual cycle and found an interaction between the cycle phase and a genetic polymorphism in the catechol-O-methyltransferase (COMT) gene, the Val^158^Met polymorphism. This polymorphism determines the activity of the COMT enzyme in that carriers of the *met/met* allelic variant have decreased enzymatic activity in the prefrontal cortex and therefore higher DA levels as compared to individuals with the *val/val* genotype (e.g., Cai and Arnsten, 1997; Weinberger et al., 2001; Gibbs and D'Esposito, 2005). In their study Jacobs and D'Esposito (2011) found that *val/val* subjects showed increased performance during the FP when E2 reaches peak level, while subjects with the *met/met* genotype performed better during menses when E2 is low. Since DA function follows an inverted-U-shaped curve with deficient or excessive DA levels leading to less optimal DA functioning, increases of E2 during the menstrual cycle might evoke different effects depending on the “baseline” DA function of an individual (for an overview see also Colzato and Hommel, 2014). This interaction between the COMT Val^158^Met polymorphism and the effect of E2 on DA related cognitive processes has recently also been observed in the context of delay discounting (Smith et al., 2014). In this study subjects showed a reduced bias for sooner/smaller rewards in the FP compared to menses, an effect mainly driven by carriers of the *val/val* genotype.

Taken together, our findings provide initial evidence for an effect of menstrual cycle phase on the preference for Go over NoGo learning in a response time adjustment paradigm. During the FP in the presence of high E2 levels and thus presumably elevated DA levels, NoGo learning was impaired and therefore the ability to slow down and to wait patiently was impeded. During this cycle phase, effective adaptation to slow speed might have been achieved by (1) increased performance monitoring during feedback processing in the RCZ and IFJ and (2) decreased reward-related responses of the VMPFC in order to suppress the initial urge to respond rapidly and to counteract the phase-specific Go bias. The fact that we found no corresponding brain-behavior correlations during the LP emphasizes the absence of the need for compensation in the presence of high PROG levels that should act positively on the NoGo pathway. In summary, these data suggest a cycle dependent modulation of temporal decision making requiring Go and NoGo learning systems.

Our results also add further evidence to the more and more described neuroregulatory effects of E2 and PROG on dopamine-related behaviors. A growing number of studies focused on endogenous variations of E2 and PROG during the menstrual cycle and reported cycle-dependent brain activation, for instance enhanced reward processing during the FP (Caldú and Dreher, 2007; Dreher et al., 2007). Moreover, cycle phase has been shown to affect working memory (Gasbarri et al., 2008; Jacobs and D'Esposito, 2011) and inhibitory control (Colzato et al., 2010). Neuroendocrinological research has only recently begun to investigate naturally occurring differences in dopaminergic transmission by taking into account hormonal shifts during the menstrual cycle. Hence, our study contributes important insights into the linkage between estradiol induced dopamine increases and their impact on temporal decision making and response time adaptation.

### Conflict of interest statement

The authors declare that the research was conducted in the absence of any commercial or financial relationships that could be construed as a potential conflict of interest.
