# Supplementary material for: How to be patient. The ability to wait for a reward depends on menstrual cycle phase and feedback-related activity
Source: Front Neurosci. 2014 Dec 9;8:401. doi: 10.3389/fnins.2014.00401 (PMC4260677; doi:10.3389/fnins.2014.00401)
Supplement: Supplementary file 1 [file DataSheet1.PDF]

Frontiers in Neuroscience
Neuroendocrine Science
Research Topic: "From Sex Differences in Neuroscience to
a Neuroscience of Sex Differences: New Directions and Perspectives"

Supplementary Material

How to be patient. The ability to wait for a reward depends on menstrual cycle phase and feedback-related activity.

Luise Reimers[1], Christian Büchel[2], Esther K. Diekhof[1]*

[1]*Neuroendocrinology Unit, Institute for Human Biology, Biocenter Grindel and Zoological Museum,University of Hamburg, Hamburg, Germany*

[2]*Department of Systems Neuroscience, University Medical Center Hamburg-Eppendorf, Hamburg, Germany*

*** Correspondence:** Esther K. Diekhof, Institute for Human Biology, Biocenter Grindel and Zoological Museum, University of Hamburg, Martin-Luther-King Platz 3, D-20146 Hamburg / Germany
Tel.: +49-40-42838-3931, Mail: Esther.diekhof@uni-hamburg.de

1. Supplementary Figure

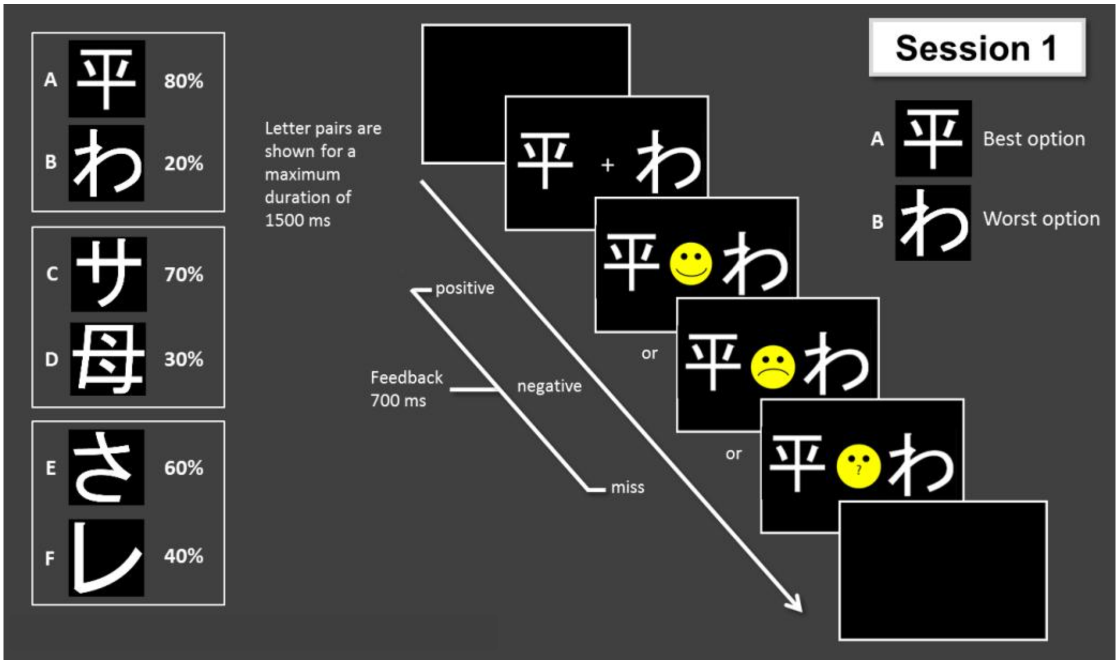

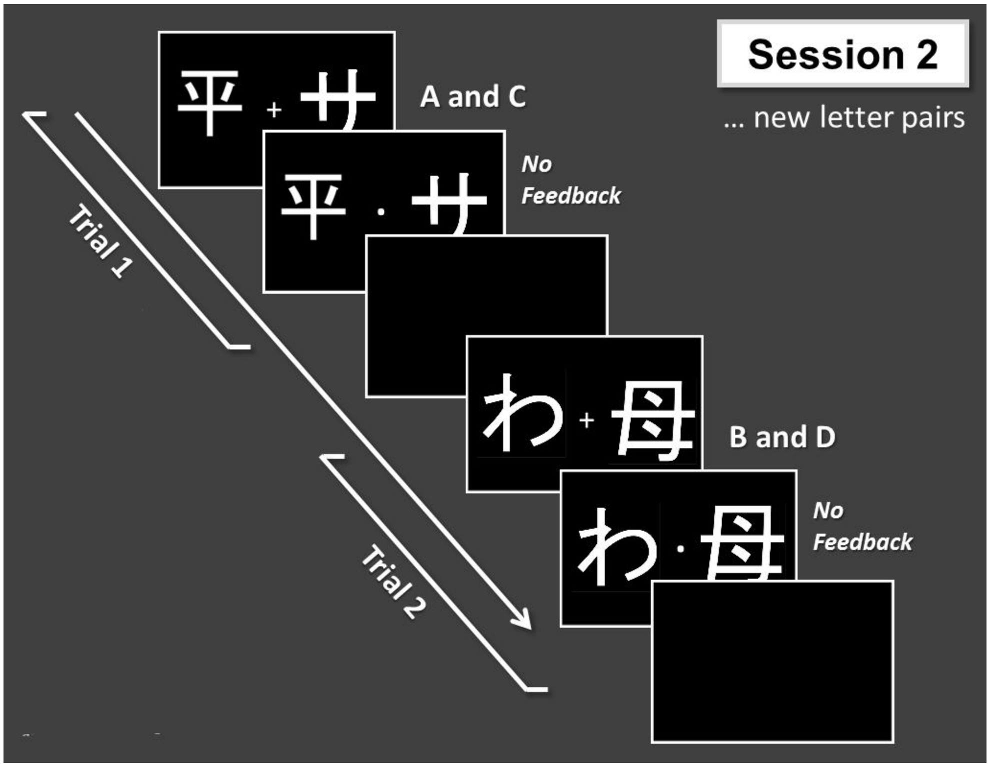

Supplementary Figure 1: the probabilistic learning task. This task (adapted from Frank et al., 2004) examined whether a good learning performance was achieved by either high reward sensitivity or by avoidance of negative outcomes. To test this, subjects were presented with three different symbol pairs (here labelled as letter pairs 'AB', 'CD', and 'EF') during the first session and asked to select the rewarded option. They received probabilistic feedback in form of smileys or grumpies according to their choice. Throughout this first learning session, the stimulus pairs contained the same letters. Accordingly, at the end of the first session subjects should have learned that the letter 'A' was the most rewarded option (with a reward probability of 80%) and that the letter 'B' was the least rewarded option (probability of negative feedback 80%). In the second session the letters were mixed into new pairs ('AC', 'AD', 'AE', 'AF', 'BC', 'BD', 'BE', 'BF', 'CD', 'CE', 'CF', 'DE', 'DF') and subjects were again instructed to select the most rewarded option. During this test session no feedback was given. The percentage of how often a subject chose the letter 'A' from the new stimulus combinations gave a measure of reward sensitivity (i.e., Go learning). On the other hand, the frequency of avoiding the letter 'B' in the new letter pairs indicated punishment sensitivity (i.e., NoGo learning). See Frank et al. 2004 for further details regarding this paradigm.

2. References

Frank, M. J., Seeberger, L. C., O'Reilly, R. C. (2004). By carrot or by stick: cognitive reinforcement learning in Parkinsonism. *Science* 306, 1940-1943. doi: 10.1126/science.1102941
